# The Antimicrobial Photodynamic Therapy in the Treatment of Peri-Implantitis

**DOI:** 10.1155/2016/7692387

**Published:** 2016-06-26

**Authors:** Umberto Romeo, Gianna Maria Nardi, Fabrizio Libotte, Silvia Sabatini, Gaspare Palaia, Felice Roberto Grassi

**Affiliations:** ^1^Department of Oral and Maxillofacial Sciences, “Sapienza” University of Rome, Via Caserta 6, 00161 Rome, Italy; ^2^University of Bari, Piazza Umberto I, 70121 Bari, Italy

## Abstract

*Introduction*. The aim of this study is to demonstrate the effectiveness of addition of the antimicrobial photodynamic therapy to the conventional approach in the treatment of peri-implantitis.* Materials and Methods*. Forty patients were randomly assigned to test or control groups. Patients were assessed at baseline and at six (T1), twelve (T2), and twenty-four (T3) weeks recording plaque index (PlI), probing pocket depth (PPD), and bleeding on probing (BOP); control group received conventional periodontal therapy, while test group received photodynamic therapy in addition to it.* Result*. Test group showed a 70% reduction in the plaque index values and a 60% reduction in PD values compared to the baseline. BOP and suppuration were not detectable. Control group showed a significative reduction in plaque index and PD.* Discussion*. Laser therapy has some advantages in comparison to traditional therapy, with faster and greater healing of the wound.* Conclusion*. Test group showed after 24 weeks a better value in terms of PPD, BOP, and PlI, with an average pocket depth value of 2 mm, if compared with control group (3 mm). Our results suggest that antimicrobial photodynamic therapy with diode laser and phenothiazine chloride represents a reliable adjunctive treatment to conventional therapy. Photodynamic therapy should, however, be considered a coadjuvant in the treatment of peri-implantitis associated with mechanical (scaling) and surgical (grafts) treatments.

## 1. Introduction

Peri-implant disease may be defined as a pathologic condition including inflammatory and other kinds of lesions affecting the soft and/or hard tissues surrounding a dental implant [1].

Peri-implantitis is characterized by a severe inflammatory process involving both mucosa and bone around the implant [2]. This represents the most diffuse cause of long-term implant failure. Bone destruction, peri-implant pockets, bleeding on probing, the possible presence of exudate, and loss of supporting tissue are involved in peri-implantitis [3].

Peri-implantitis is due to bacterial contamination or technical problems, related to the implant surface itself or to implant support placement and the subsequent osseointegration process. Osseointegration may be influenced by mistakes or complications occurring in the surgical phase or masticatory overload.

The bacterial biofilm on the implant surfaces is similar to the one in periodontal disease. The microflora includes microorganisms such as* Aggregatibacter actinomycetemcomitans*,* Peptostreptococcus micros*,* Campylobacter rectus*,* Capnocytophaga* spp.,* Porphyromonas gingivalis*, and* Tannerella forsythia*. However, it should be stressed that the residual teeth could influence the composition of microflora. Bacterial species observed in edentulous patients differ from those of partially edentulous subjects. On this basis, the idea that the presence of bacteria involved in periodontal disease could contribute to development of peri-implantitis seems to be plausible [4].

During the surgical stage, the treatment in the initial stage included elimination and of plaque and calculus, decontamination of the implant surface, and maintenance of healthy conditions [5].

Decontamination of implant surfaces is a challenging goal. Several different treatments have been proposed in the literature [6, 7]. Cleaning the surfaces can be through mechanical (dental curettes, ultrasonic scalers, and air-powder abrasive) and chemical (citric acid, H_2_O_2_, chlorhexidine digluconate, and EDTA) procedures, in association with local or systemic antibiotics [8, 9].

Lasers can be used in decontamination of implant surfaces. The most frequently used include diode, erbium lasers, and CO_2_ due to their hemostatic properties, selective calculus ablation, and bactericidal effects [10].

An alternative approach to dental implant decontamination is the association of the conventional treatment with photodynamic therapy (PDT).

Photodynamic therapy includes the use of a low-power diode laser in combination with photosensitizing compounds. These components are linked to the bacterial membrane and, when excited, react with the substrate. The photosensitizer binds to the target cells and when it is irradiated with light of specific wavelength, in the presence of oxygen, it undergoes a transition from a low-energy ground state to an excited singlet state; then singlet oxygen and other very reactive agents are produced, which are toxic to these target cells [11].

Photodynamic therapy (PDT) has received more attention in dentistry in recent years. The application of photosensitive dyes into pockets and their activation with light promote killing of periodontal pathogens. Outcomes of clinical studies in subjects with chronic periodontitis show beneficial effects of PDT on the reduction in gingival inflammation [12].

The effects of PDT on the treatment of ligature-induced peri-implantitis were investigated in dogs. The results revealed a reduction in bacterial counts of* Prevotella intermedia*/*nigrescens*,* Fusobacterium* spp., and beta-haemolytic* Streptococcus* [13].

Several studies have demonstrated bacteria destruction can be achieved without any damage to the treated titanium surfaces [14].

The aim of this experimental study is to demonstrate the efficacy of antimicrobial photodynamic therapy in addition to the traditional approach.

## 2. Materials and Methods

40 subjects were involved in the study ranging in age from 34 to 68 years, referred to the Periodontology Department of the Dentistry Unit at Bari University Hospital. The subjects had given their consent to treatment. The study was conducted following the Declaration of Helsinki, according to the local Ethical Committee.

The patients were selected with these inclusion criteria: overall plaque index (PlI) ≥40% and at least one implant site with the following characteristics: probing depth (PD) ≥4 mm, bleeding on probing (BOP), and presence of suppuration. A full mouth series for each patient was performed to confirm diagnosis. Six sites for each implant were analyzed.

Exclusion criteria included decompensated systemic disease, degenerative bone disease, chronic immune-based mucomembranous disorders (e.g., lichen planus, pemphigoid, pemphigus, and systemic lupus erythematosus), chemotherapy or radiotherapy to the head and neck area, pregnancy, presence of teeth with periodontitis adjacent to sites affected by peri-implantitis, implants placed in fresh extraction sockets, smoking >10 cigarettes daily, and alcoholism.

The null hypothesis was that nonstatistically significant differences are observed with respect to the clinical parameters (e.g., PPD, BOP, and PlI) between the two treatment modalities (i.e., adjunctive PDT test group versus control group).

The primary outcome variable was the reduction of PD in peri-implant sites with probing depth ≥4 mm. Secondary outcome variables were the changes in BOP and PlI.

The ratio of this study was based on the capacity of photodynamic therapy to promote bacterial inactivation by light and not by heat. This is achieved with 40-milliwatt laser beam power, with no heat being developed. 360° light irradiation is obtained by means of special probes ensuring optimal light beam diffusion.

123 dental implants were analyzed. The patients were randomly assigned to two groups, that is, a test group (63 implants) and a control group (59 implants), using a software to create a randomization list (https://www.random.org/) and assigning a code to each patient.

For both groups of patients the following indices were measured by means of a plastic probe: the plaque index (PlI), based on the Plaque Control Record (PCR, [15]), bleeding on probing (BOP) with or without suppuration, and probing depth (PD).

**Figure 1:**
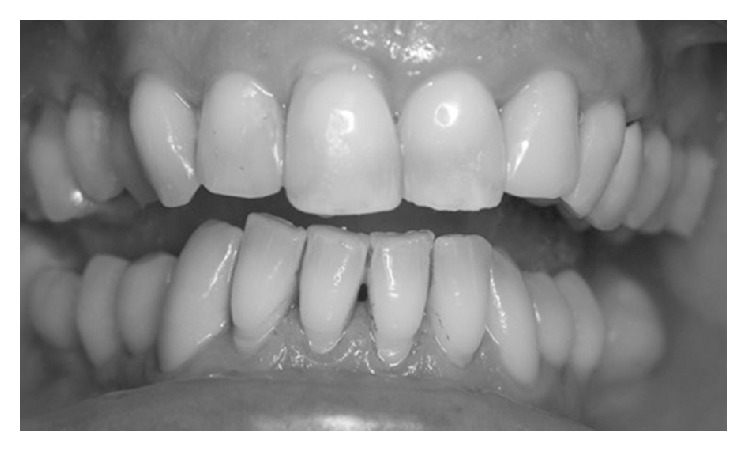
Ultrasonic debridement has been performed.

Mechanical and manual decontamination of the oral cavity was performed using air polishing with micronized glycine powder to remove plaque and discolorations and expose the underlying calculus (Figure 1). The latter was removed with a piezoelectric ablator in combination with a universal tip for the scaling of natural teeth and a special nonmetal tip for implant scaling. Root debridement at sites with PD ≥4 mm was performed with a periodontal ultrasonic unit and implant debridement at sites with PD ≥4 mm was done with carbon-fiber-reinforced plastic curettes.

At the end of the procedure, according to the code of the envelope, the dental hygienist considered in the test group the addition of laser-assisted antimicrobial photodynamic therapy based on the HELBO Protocol at implant sites with PD ≥4 mm.

The treatment of PDT was performed using HELBO TheraLite (Bredent medical), diode laser battery powered with a wavelength of 670 nm and output of 75 mW/cm^2^, with a spot size of 0.06 cm in diameter. HELBO Blue photosensitizer was used, a liquid containing methylene blue (methylthioninium hydrochloride, also known as 3,7-bis phenothiazine-5-ium chloride). The concentration of photosensitizer was 10 mg/mL with absorbance peak at 670 nm. Its use as a chromophore in photodynamic therapy is justified by its relative stability in the light, which makes it an important generator of singlet oxygen (ET = 142.1 kJ/mol with ΦΔ = 0.60 in water).

**Figure 2:**
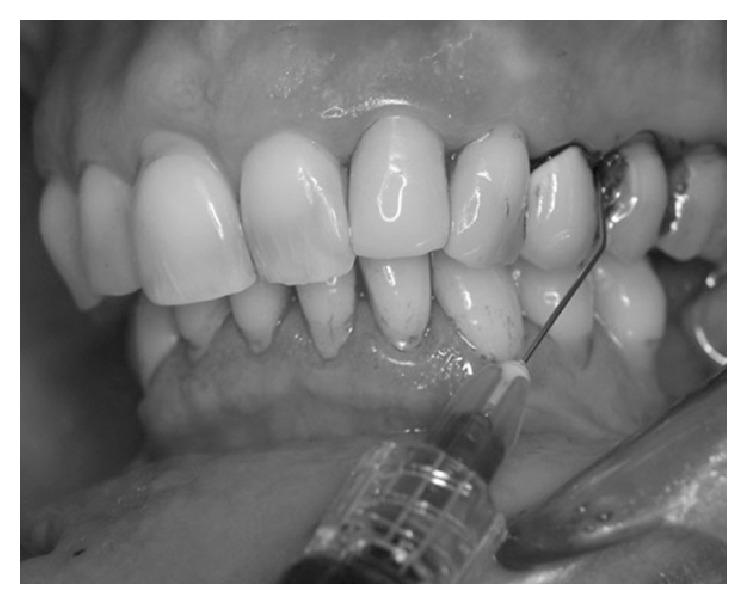
The special HELBO® Blue Photosensitizer is applied within the peri-implant pocket starting from the bottom.

**Figure 3:**
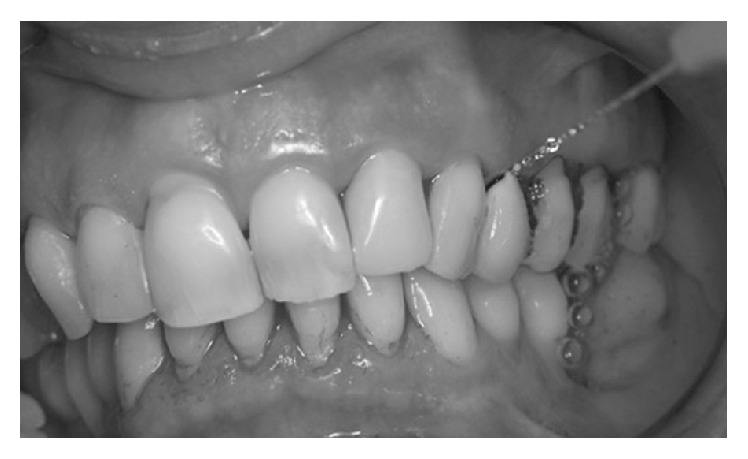
Rinsing the fluid off the pocket.

**Figure 4:**
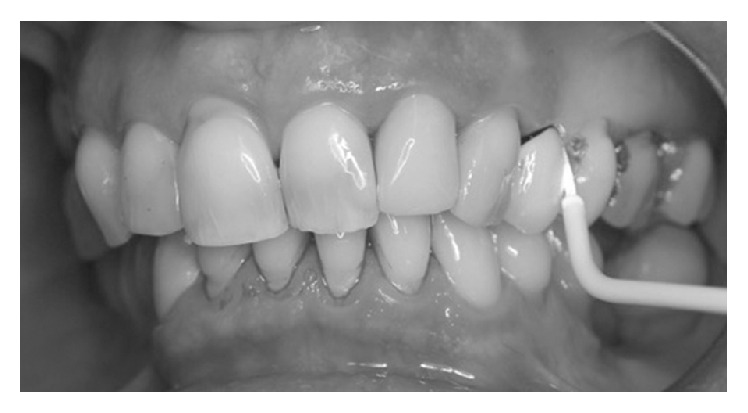
Exposure to HELBO TheraLite diode laser for about 1 minute of the implant surface.

The photosensitizer was applied inside the peri-implant pocket starting from the bottom and moving in apical-coronal direction (Figure 2). Care was taken to avoid the formation of air bubbles, allowing the fluid to dye all bacteria by leaving it in situ for 60 seconds. After rinsing the fluid off the pocket and suctioning excess liquid (Figure 3), the previously dyed implant surface was exposed to HELBO TheraLite diode laser for 1 minute (Figure 4). The fluence was 25.54 J/cm^2^, while the total energy applied was 1592 J/cm^2^. TheraLite illumination was applied using circular movements. This type of movement promotes the best activation of the dye molecules with the laser light and transfers their energy to local oxygen. The resulting singlet oxygen is highly aggressive and capable of destroying bacterial cells.

Both groups of patients received home oral hygiene instruction. They were advised to brush their teeth for two minutes, twice a day, using an oscillating-rotating electric toothbrush with little toothpaste and a special brush for interproximal hygiene.

*T1 (6 Weeks)*. In both groups the same clinical measurements were taken as those at baseline and home oral hygiene instruction was provided again.

*T2 (12 Weeks)*. In both groups the same clinical measurements were taken as those at baseline and home oral hygiene instruction was provided again. This was followed by a deplaquing session with glycine air polishing.

**Figure 5:**
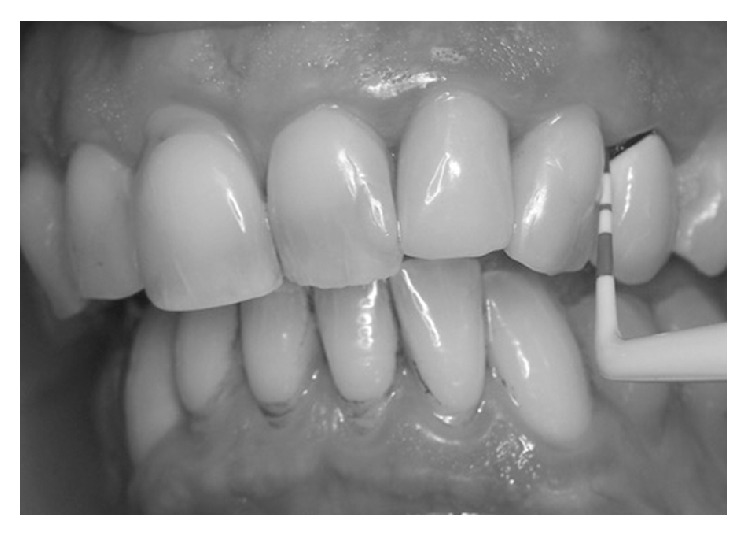
Final probing.

*T3, End of the Study (24 Weeks)*. The same clinical measurements were taken as those at baseline (Figure 5).

A weighted arithmetic mean was taken to calculate average values for each group in terms of PD, BOP, and PlI at 6, 12, and 24 weeks using a computer software (Graph Pad Prism 5®).

## 3. Results

As early as at the 6th week of the study, reductions in clinical parameters were observed in both groups compared with baseline values. The reductions were more marked in the test group.

PD average values were calculated. Average values were lower than the baseline. The reduction was first seen as early as at 6 weeks, to be confirmed at 12 weeks, when the values further declined. The readings remained constant at 24 weeks. Test group showed a better value of PD, with an average value of 2 mm if compared with control group (3 mm).

With regard to the plaque index, average value was calculated for each group. In this case, a significant score reduction was recorded as early as at the 6th week. Despite improving of daily oral hygiene practices, the plaque index variations were not constant. Test group showed a PlI of 17% after 24 weeks. Control group showed a PlI of 25%. There were no significant differences between the two groups (Table 2).

Regarding BOP, at baseline, all patients had bleeding on probing and suppuration at the peri-implant sites under investigation. In the test group patients, these signs of inflammation had gradually improved to disappear completely by the 24th week. In the control group, however, some improvements were recorded, but not all of the patients achieved complete remission (Table 3).

## 4. Discussion and Conclusion

Peri-implantitis has been defined as an inflammatory process that affects the soft tissues surrounding an osseointegrated implant in function with concomitant loss of supporting marginal bone. Peri-implant mucositis, in contrast, is a reversible inflammatory reaction of the mucosa adjacent to an implant without bone loss. Colonization of oral implant surfaces with bacterial biofilms occurs rapidly and the biofilm development seems to play an important role in altering the biocompatibility of the implant surface and, thus, enhancing peri-implant disease development [16].

Since photodynamic therapy has been introduced in dentistry, several advantages of laser and PDT in the many fields of dentistry have been described in the literature. An increasing interest is recently growing regarding PDT in implant dentistry and as a coadjuvant treatment for peri-implantitis [17]. It employs visible light (laser) and a dye (photosensitizer), the combination of which leads to the release of free oxygen radicals, which in turn can selectively destroy bacteria and their products. Although PDT has been used in the field of medicine since 1904 for light-induced inactivation of cells, microorganisms, and molecules, Branemark's discovery of osseointegration in 1965 was extremely important to restorative treatments and, particularly, functional oral rehabilitation. A large number of patients have been rehabilitated with dental implants, and, consequently, more cases of success and failure have appeared over the years. Thus, peri-implantitis has become an increasingly frequent problem in dentistry.

Laser therapy has some advantages in comparison to traditional therapy. It is well known that laser has the ability to modify dentin so as to obtain the exposition of collagen fibers. The exposition of collagen may facilitate the attachment of blood clot and its stabilization. This, in turn, may favor a speedy healing and the obtainment of a new collagen attachment in spite of long junctional epithelium. This fact could explain the faster and greater healing of the wound and the results in the test group. It is clear that further histological analysis should be carried out to demonstrate this idea [18].

Thus, photodynamic therapy (PDT) may be one such treatment alternative. Only in the last 10 years or so clinical studies have examined its application in the oral cavity. The current data show that treating chronic periodontitis with PDT alone versus conventional SRP treatment has no additional benefit [19]. In contrast, combining PDT and SRP does provide an additional benefit, particularly in lesions with unfavorable anatomic conditions. A clinical controlled study compared the effect of PDT alone (without subgingival SRP) with SRP in the treatment of aggressive periodontitis [20, 21].

In addition to this, during peri-implantitis treatment, HELBO technology offers the advantage of a noninvasive, painful, rapid bacterial inactivation thanks to liberation of oxygen. Oxygen allows the destruction of bacteria membrane, and on the other hand its sparkling effect permits dangerous enzymes and collagenosis to be quickly removed from the pocket, for a better bacterial removal and, as a consequence, could facilitate healing.

The improvement of values analyzed was more marked in the test group (Table 1). Test group showed a better value of PD, with an average value of 2 mm if compared with control group (3 mm).

Regarding PlI the significant reduction recorded at the 6th week was followed by a slight increase at 12 weeks, with values remaining constant up to the 24th week. However, the plaque index score for each patient at 24 weeks was anyway lower than at baseline.

Finally, a comparison between baseline and final average bleeding on probing (BOP) and suppuration values also shows substantial improvement.

Thus, the results obtained in this study suggest that photodynamic therapy could be considered an effective method for bacterial reduction on implant surfaces [17–22].

Our study also confirms its effectiveness in reducing clinical indices and the bacterial load at sites affected by peri-implantitis, with significant bacterial detoxification being achieved.

Photodynamic therapy should, however, be considered a coadjuvant in the treatment of peri-implantitis and associated with mechanical (scaling) and surgical (grafts) treatments in order to control peri-implant disease.

## Figures and Tables

**Table 1:** Probing depth average values in test and control group after 6, 12, and 24 weeks.

PD	Test	Control
Baseline	5 mm	5 mm
6 weeks	3 mm	3 mm
12 weeks	2 mm	2 mm
24 weeks	2 mm	3 mm

**Table 2:** Plaque index values in test and control group after 6, 12, and 24 weeks.

PlI	Test	Control
Baseline	60%	62%
6 weeks	11%	12%
12 weeks	17%	21%
24 weeks	17%	25%

**Table 3:** BOP and suppuration values in test and control group after 6, 12, and 24 weeks.

BOP/suppuration	Test	Control
Baseline	100%	100%
6 weeks	20%	35%
12 weeks	10%	20%
24 weeks	0%	10%
